# Extent of Tissue Washing Can Significantly Alter the Composition of Adipose-Derived Stromal Vascular Fraction Cell Preparations: Implications for Clinical Translation

**DOI:** 10.1093/stcltm/szad025

**Published:** 2023-06-03

**Authors:** Gabriela Aguilo-Seara, William Molair, Hulan Shang, Scott Northrup, Joshua A Grosser, Ramon Llull, Adam Katz

**Affiliations:** Department of Plastic Surgery, Wake Forest School of Medicine, Winston-Salem, NC, USA; Department of Plastic Surgery, Wake Forest School of Medicine, Winston-Salem, NC, USA; Department of Plastic Surgery, Wake Forest School of Medicine, Winston-Salem, NC, USA; Department of Plastic Surgery, Wake Forest School of Medicine, Winston-Salem, NC, USA; Department of Plastic Surgery, Wake Forest School of Medicine, Winston-Salem, NC, USA; Department of Plastic Surgery, Wake Forest School of Medicine, Winston-Salem, NC, USA; Department of Plastic Surgery, Wake Forest School of Medicine, Winston-Salem, NC, USA

**Keywords:** cell- and tissue-based therapy, transplants/grafting, lipectomy/liposuction, cell separation, cell survival, guided tissue regeneration

## Abstract

Stromal vascular fraction (SVF) cell preparations have recently attracted much interest as a form of autologous cell therapy. These heterogenous cell populations typically include some proportion of blood-derived cells (BDCs)—including both red blood cells (RBCs) and leukocytes (WBCs). The objectives of this paper were to evaluate the effects of tissue washing and hypotonic RBC lysis—separately and together—on BDC concentrations within SVF, and further to explore whether BDCs can confer detectable and modifiable effects on adipose-derived cell activity. Using various cell culture assays, flow cytometry and ELISA analysis of human-derived SVF preparations, we show that thorough washing of adipose tissue prior to enzymatic dissociation effectively removes RBCs from SVF preparations as well as standard lysis methods and significantly alters the type and relative quantities of WBCs. In addition, these studies demonstrate that potentially toxic RBC components are detectable for up to 1 week in cultures containing RBC lysate, but not those with intact RBCs, and, that culture-expanded cells proliferate significantly more in the presence of intact RBCs versus RBC lysis products or control media. Broadly, these data exemplify how different seemingly mundane tissue processing steps can significantly influence SVF identity/composition, purity, and potency. Based on the findings of this work, we propose that translational efforts in the field would benefit by a better understanding of the impact of RBCs, WBCs, and non-viable cells on the in vivo therapeutic activity of SVF therapies.

## Graphical Abstract

**Graphical Abstract:**
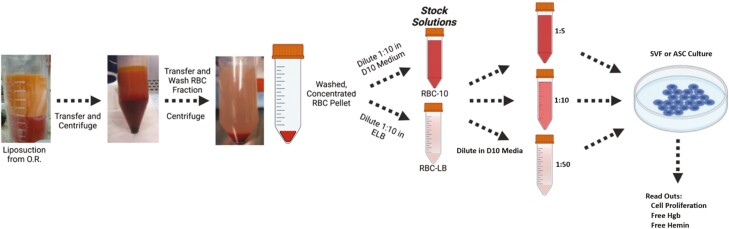

**Significance Statement**

In this paper, we show how washing and/or red blood cell lysis of lipo-harvested adipose tissue can effect the cell composition and inflammatory properties of stromal vascular fraction (SVF) cells prepared for autologous cell therapies. Given that autologous adipose-derived SVF cell therapies have entered late stage clinical testing, these findings are significant in that they shed some light on why cell-based therapies may perform differently between individual surgeons or preparation techniques, a topic that has been reported on extensively but scarcely researched.

## Introduction

Autologous, point-of-care cell therapies using adipose-derived stromal vascular fraction (SVF) cells have advanced to late-stage clinical trials for a growing number of indications.^1-4^ Although no FDA-approved adipose-derived cell therapies exist at this time several platforms, devices, and methods are in development to enable this point-of-care treatment paradigm which circumvents the cost, complexity, and time-delay associated with cell culture. While most in the field understand that there is inherent variability between, and heterogeneity within SVF cell “product” compositions, it may be easily assumed that largely similar tissue and cell processing methods will yield SVF preparations with similar composition and activity. However, careful evaluation of the literature suggests otherwise.

Harvested-adipose tissue contains blood that normally resides within the vasculature as well as that which extrudes into the extravascular space due to trauma and bleeding. The number/volume of blood-derived cells (BDCs, ie, red blood cells (RBCs) and/or white blood cells (WBCs)) that exist in the initial post-digestion SVF pellet is directly related to the extent of tissue washing after harvest but before enzymatic digestion. BDCs that remain after washing and survive enzymatic dissociation are inevitably concentrated into the initial SVF cell pellet. Although the majority of SVF isolation methods reported in the literature describe the subsequent “removal” of RBCs using one of several available lysis techniques, leukocytes are not affected by this approach. There is a significant gap in the literature regarding how SVF preparation steps might impact the presence of these “marginalized” cell types (ie, RBC and WBC) within SVF preparations and how, in turn, such might impact SVF bioactivity.

For instance, the percentage of WBCs within SVF preparations varies widely in the literature from 2.5% to 53% and nonviable cells may account for another 10%-30% depending on the methods used.^5-8^ Similarly, RBCs may exist within SVF preparations to varying amounts, yet the extent of RBC “contamination” within SVF preparations is almost never reported in the literature. Most protocols remove RBCs with an erythrocyte lysing buffer (ELB) step as part of “standard” SVF isolation procedures, and it may be assumed that this reduces RBC content to negligible levels. However, even if this is the case, the lysis of RBCs releases free hemoglobin and other toxic substances into the extracellular space—or in this case, within the SVF pellet. Free extracellular hemoglobin and its byproduct hemin are capable of causing significant toxicity and damage that is dependent on the duration, quantity, and tissue localization of the exposure—the effects of which may be cumulative.^9^ It is, therefore, conceivable that the act and/or extent of RBC lysis during SVF preparation results in a pro-inflammatory milieu that negatively impacts SVF activity.

In the present paper, we first examine SVF and ASC proliferation in the presence of intact and lysed RBCs and quantify the effect of RBC lysis on hemoglobin and hemin concentrations in cell media. We then utilize flow cytometry to compare the effects of tissue washing and hypotonic RBC lysis, separately and together, on BDC concentrations within SVF. Our central hypothesis is that the extent of initial washing of lipo-aspirated adipose tissue can significantly impact the final composition of SVF preparations and that BDCs can confer detectable and modifiable effects on adipose-derived stromal cell activity.

## Methods

### Tissue Collection

Lipoaspirate (LA) tissue was collected from surgical patients undergoing elective liposuction under an IRB-approved protocol. Tissue was processed the day of collection.

### Preparation of RBC- and Lysed-RBC Conditioned Media

**Figure 1.**
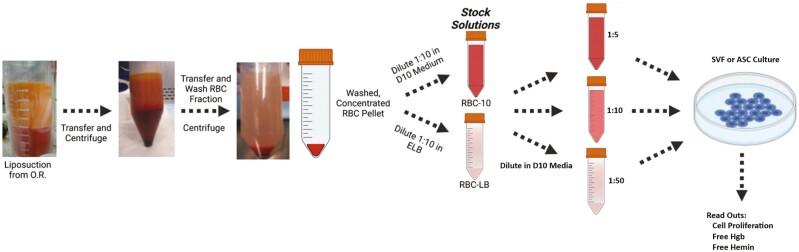
Preparation of intact RBC and lysed RBC conditioned media. intact RBCs or lysed RBCs were prepared in D10 culture media at 3 different concentrations and then added to SVF or ASC cultures. ELB: erythrocyte lysing buffer.

Human LA was centrifuged at 600 *g* for 10 minutes to separate the sample into oil, fat, water, and RBC layers. The RBC pellet was collected and washed 3 times. Three different dilutions of RBCs were prepared from the resulting pellet by suspending in equal volumes of either Dulbecco’s modified eagle’s medium (DMEM/F12) with 10% FBS and 1× Antibiotic-Antimycotic (Gibco Amarillo TX) (D10) or ammonium-chloride-potassium (ACK) lysing buffer. RBCs undergoing lysis were then centrifuged, and the supernatant was diluted in D10 in a similar fashion (Fig. 1).

### SVF and ASC Isolation and Culture

Human LA was collected in sterile LA containers, transferred, and centrifuged at 1200 *g* for 3 minutes to separate the sample into oil, fat, water, and RBC layers. Oil, water, and RBC layers were removed via serological pipette, and the resulting fat layer was washed with Lactated Ringer’s solution (LR) and centrifuged at 1200 *g* for 3 minutes. The remaining water, RBC, and oil layers were then removed by pipette. This washing process was repeated 2 more times. Next, the resulting fat tissue was incubated 1:1 with 200 CDU/mL Collagenase Type 1 (Worthington Biochemical, Lakewood NJ) dissolved in LR in a heated shaker set to 41 °C and 350 *g* for 45 minutes. The resulting solution was centrifuged at 600 *g* for 10 minutes allowing the formation of a top oil layer, an adipocyte layer, a water layer, and a bottom enzymatic SVF (eSVF) layer. The bottom layer was pipetted off, strained through 500 and 100 µM cell strainers, counted, then placed into tissue culture dishes or plates and immediately covered with culture media. These were then placed into an incubator set to 37 °C and controlled at 5% CO_2_ for culture.

Freshly isolated SVF cells and second passage ASCs were cultured in the media solutions found in Table 1 for up to 7 days on 96-well culture plates replicated 8 times. Cell-free conditioned medium was collected on days 0, 3, and 6/7 and evaluated for RBC-derived hemin and hemoglobin using ELISA. Cell proliferation was evaluated after 6 or 7 days using PrestoBlue (Fluorescence method) (Thermo Fisher Walton, MA).

**Table 1.** Culture solutions and their components.

Culture solution		Additive	Diluent
1	RBC—10 1:5	Intact RBCs	D10 medium
2	RBC—10 1:10	Intact RBCs	D10 medium
3	RBC—10 1:50	Intact RBCs	D10 medium
4	RBC—LB 1:5	Lysed RBCs	D10 medium
5	RBC—LB 1:10	Lysed RBCs	D10 medium
6	RBC—LB 1:50	Lysed RBCs	D10 medium
7	ELB 1:5	Lysis buffer	D10 medium
8	ELB 1:10	Lysis buffer	D10 medium
9	ELB 1:50	Lysis buffer	D10 medium
10		—	D10 medium

Culture solutions were created from either whole RBCs, lysed RBCs, or elution buffer. D10 Medium was used as the diluent for all culture solutions. These culture conditions were designed to simulate an SVF graft that had either been exposed to ACK-based RBC lysis, washing, or direct implantation with different amounts of RBC contamination present in each of them.

### Presto Blue Assay for Cell Proliferation

PrestoBlue dye was purchased from ThermoFisher (Cat#P50201) and used to quantify viable SVF cells at aforementioned timepoints. Absorbance was measured using an Infinite M200 Plate reader (Tecan Life Biosciences, Männedorf Switzerland). These results were then normalized to ASCs grown in D10 as a control.

### ELISA Quantification of Hemin and Hemoglobin

ELISA kits were purchased for Hemin (Sigma-Aldrich, Cat# MAK316) and Hemoglobin (Invitrogen, Cat# EIAHGBC) and stored at 4 °C until use. Test samples were collected in Eppendorf tubes and mixed well, then stored in −80 °C freezers until analysis. Samples were then evaluated per kit instructions. For Hemin, Heme calibrators and samples were all run in duplicate, with all samples falling within the calibrator range. After a 5-minute incubation at room temperature, the absorbance was measured at 400 nm in an Infinite M200 plate reader (Tecan Life Biosciences, Männedorf Switzerland). For hemoglobin, sample and hemoglobin standards were run in duplicate. Following a 30-minute incubation at room temperature, the absorbance was measured at 570 nm in an Infinite M200 plate reader (Tecan Life Biosciences, Männedorf Switzerland). All diluted samples fit within the standard curve with *R*^2^ > .999.

### Adipose Tissue Preparation and Washing

Human LA was obtained as previously described by collection in sterile containers. After transfer to the lab, the LA was agitated to create a uniformly mixed sample and then quickly transferred in equal amounts to 2 separate GID SVF-2 devices.^1^ In one device, lipo-effluent was allowed to layer by gravity sedimentation for 5 minutes, and the dependent layer of aqueous solution was then removed by sump drain aspiration. This approach was designated as “unwashed” tissue. Effluent in the second device was washed using repeated cycles of agitation and sump drain removal of rinse solution until the effluent was clear (“full wash”). All subsequent cell isolation methods were identical between groups as per manufacturer instructions and as previously published.^10^

#### Flow Cytometry

Flow cytometry was performed using a BD FACS Canton II machine. Antibodies were purchased from BD Biosciences (Supplementary Table S1). Cells were prepared by filtering through a 70 µm filter and counting the resulting single-cell suspension. 500 000 to 1 000 000 cells were added to the bottom of flow tubes and spun at 250 *g* for 5 minutes. Cells were then washed with 500 µL 1× HBSS and centrifuged. Cells were then resuspended in 10 µL PBS and 10 µL Mouse Gamma Globulin working solution (Jackson ImmunoResearch, West Grove, PA) was added to the sample tube and incubated on ice in the dark for 15 minutes. Flow markers (BD Biosciences, Franklin Lakes, NJ) were added at 5-20 µL according to markers concentration and mixed briefly before incubating at 4 °C in the dark for 30 minutes. Samples were washed with 0.5 mL flow buffer before being resuspended in 400 µL of flow buffer prior to flow cytometry.

### Statistics

Statistics were performed using GraphPad Prism (GraphPad Software, San Diego CA) including descriptive statistics. For Presto Blue Assays and hemoglobin and hemin ELISAs, ANOVAs and Dunnett’s multiple comparison tests were performed. All flow cytometry results were analyzed using 2-tailed *t* tests.

## Results

### Patient Population and Sample Acquisition

Liposuction harvested-adipose tissue from 12 patients, 10 females and 2 males, was used for this study. The average patient age was 44.5 years old. The average BMI was 28.4, and 3 patients had BMIs in the obese range. Ten of the patients identified as Caucasian, and 2 identified as Black or African American. All fat samples were obtained by liposuction performed by 4 surgeons. Liposuction was performed with a 4-mm diameter cannula in a Mercedes configuration. Donor sites included the abdomen, thighs, flanks, and back. Tumescent volume ranged from 1050 cc to 3000 cc, with an average volume of 1967.8 cc. The total volume of liposuction obtained ranged from 200 cc to 1100 cc, with an average volume of 511.1 cc.

### Presto Blue Assay

**Figure 2.**
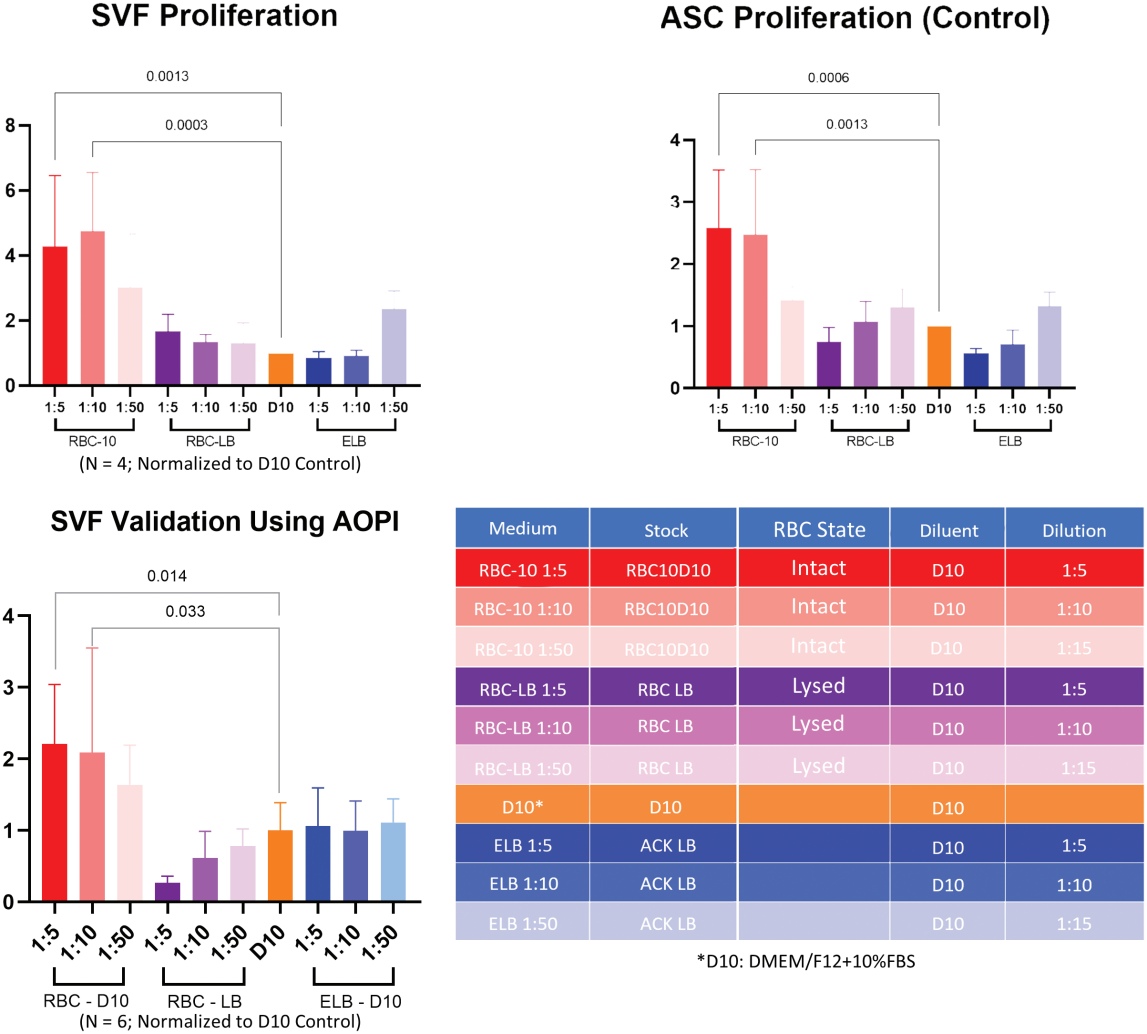
Proliferation of adipose-derived cells in media with intact RBCs or RBC lysate. Fresh SVF cells and ASCs were cultured in a variety of conditioned media as summarized above. Presto Blue Assay was used to quantify cell numbers (*n* = 4). Human SVF cells and culture-expanded ASCs proliferate significantly more in the presence of intact RBCs compared to standard D10 medium, whereas cells cultured in RBC lysate media do not.

SVF cell proliferation, measured by Presto Blue assay, was measured in 4 different media solutions, including D10 media with various concentrations of whole RBCs and lysed RBCs (ie, supernatant) (Fig. 2). D10 media alone and D10 with lysis buffer were used as controls. There was no statistically significant difference in cell proliferation between SVF samples cultured with lysed RBCs at any concentration (1:5, 1:10, or 1:50) when compared to plain media or media with lysis buffer. However, there was significantly more cell proliferation of SVF samples cultured with intact RBCs at concentrations of 1:5 (mean difference of 3.280 with CI, 1.097 to 5.463, *P* = .0013) and 1:10 (mean difference of 3.768 with CI, 1.585 to 5.950, *P* = .0003) compared to D10 media alone. The results of SVF proliferation were compared to ASCs cultured in the same media solutions. While ASCs cultured in lysed RBCs had no significant difference from D10 (control) media alone or with lysis buffer, there was a statistically significant increase in cell proliferation in ASC samples that were cultured with higher concentrations of intact RBCs (1:5 concentration, *P* = .0006; 1:10 concentration, *P* = .0013). These represented a 1.58 (CI, 0.5955 to 2.560) and 1.48 (CI, 0.4980 to 2.462) mean difference, respectively (Fig. 2). From these data, we conclude that SVF cells and culture-expanded ASCs proliferate significantly more in the presence of intact RBCs compared to standard D10 medium.

### Quantification of Hemoglobin and Hemin

**Figure 3.**
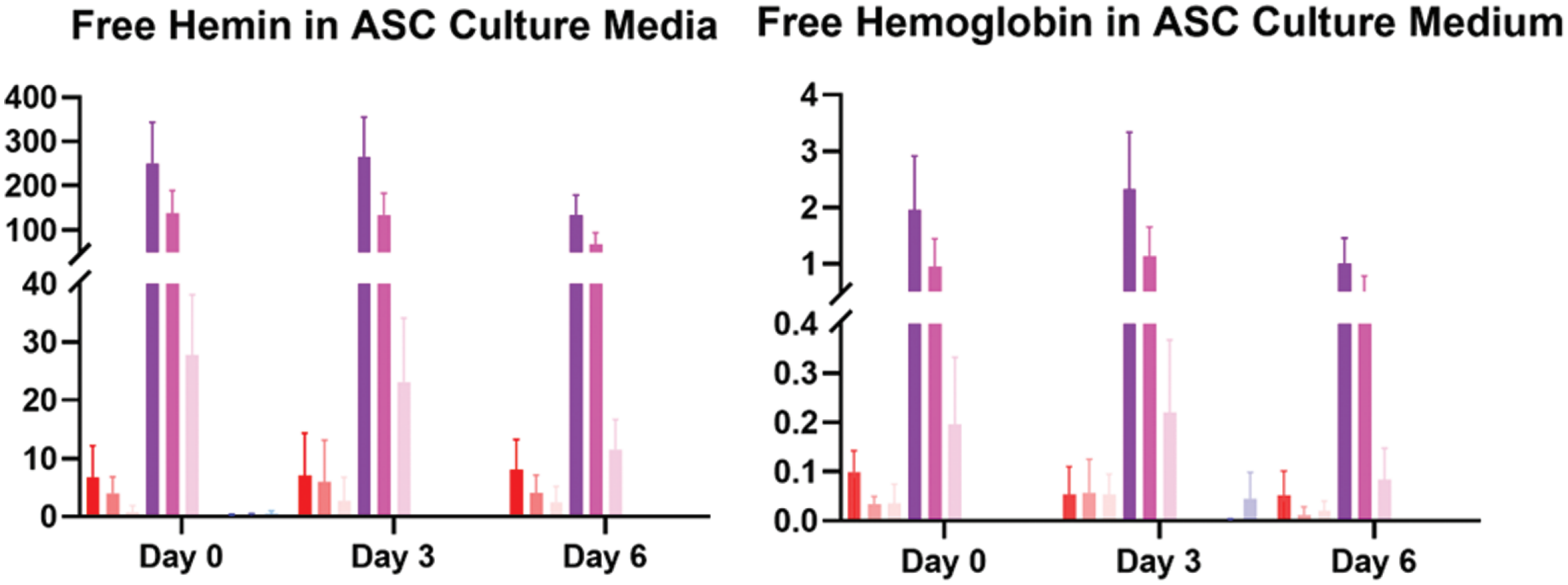
Hemin and hemoglobin concentrations in culture media over time. Both hemin and hemoglobin were significantly (*P* < .05) higher in lysed RBC media (RBC-LB) than in either control (D10) or intact RBC media (RBC-10) after 6 days of culture. Values were determined using ELISA for each specific compound (*n* ≥ 4). Based on these results, potentially toxic RBC components are detectable for up to 1 week in cultures containing RBC lysate, but not in those culture with intact RBCs.

ELISA was used to measure the amount of free hemoglobin and hemin released into the media during culture of each of these solutions. At day 0, there was no significant difference in hemoglobin or hemin levels in SVF cultures with intact RBCs, plain D10 medium, or D10 medium with lysis buffer. There was, however, a statistically significant increase in both hemoglobin and hemin in SVF cultures with lysed RBCs in the media compared to cultures in plain D10, particularly at higher concentrations. SVF cultures in 1:5 lysed RBC media solutions had a 1.95 mean difference in hemoglobin (CI, 1.260 to 2.647; *P* < .0001), and a mean difference of 250.5 in hemin levels (CI, 182.6 to 318.5; *P* < .0001). In SVF cultures with 1:10 dilution of lysed RBC media, there was a 0.95 mean difference in hemoglobin (CI, 0.2586 to 1.646; *P* = .0036) and a mean difference of 137.6 (CI, 69.52 to 205.4; *P* < .0001). These trends were consistent and highly statistically significant for both hemoglobin and hemin at days 3 and 6 of culture—despite 50% media replacement on day 3. Finally, these patterns were also observed when hemoglobin or hemin was measured in cultures of ASCs under the same conditions (Fig. 3). From these results, we conclude that potentially toxic RBC components are detectable for up to 1 week in cultures containing RBC lysate, but NOT in those with intact RBCs.

### Flow Cytometry

**Figure 4.**
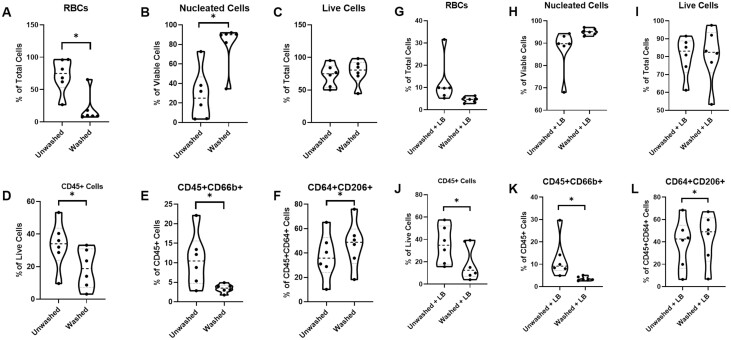
Thorough adipose tissue washing significantly reduces the number and types of blood-derived cells in SVF preparations. Flow cytometry was used to determine changes in cell populations depending on the method of purification (*n* = 6). Thorough tissue washing is as effective as ELB at removing RBCs from SVF preparations and provides advantages of time savings and diminished risk of potentially toxic substances. Thorough washing also significantly alters the type and relative quantities of WBCs in SVF preparations, significantly reducing neutrophils while increasing the percentage of M2 macrophages.

Flow cytometry was used to compare RBC, nucleated cell, and select WBC populations in unwashed samples, unwashed samples with lysis buffer, washed samples, and washed samples with lysis buffer. A summary of flow cytometry results is presented in Fig. 4. When evaluating for RBC composition of each sample, all unwashed samples contained significantly more RBCs compared to all washed samples, as well as when compared to unwashed samples treated with lysis buffer. There were no other statistically significant findings.

When evaluating for nucleated cell composition of each sample, there were significantly lower levels of nucleated cells in SVF preparations from unwashed samples compared to preparations from unwashed samples exposed to lysis buffer (mean difference of 57.89 with CI, 14.41 to 101.4; *P* = .0044), or washed samples (mean difference of 49.98 with CI, 11.98 to 87.97; *P* = .0047). There was no difference in live cell composition across any of the samples.

When looking at CD 45+ (ie, WBC) levels, all unwashed samples—whether treated with lysis buffer or not—had significantly more CD 45+ cells than washed samples. Within unwashed or washed groups, lysis buffer had no significant effect on CD 45+ levels. Evaluation of neutrophil (CD 66b+) populations also demonstrated significant differences between groups with all unwashed treatment groups containing significantly more neutrophils than washed treatments, regardless of whether lysis buffer was used or not. A similar (but opposite) pattern was found for CD 206+ cells which putatively stains M2 macrophages. For CD 206, all unwashed samples contained significantly fewer CD 206+ cells regardless of lysis buffer treatment. There were no statistically significant differences across any of the groups when evaluating for CD 64, a macrophage marker, CD 86, a M1 macrophage, or CD 14, a monocyte marker. From these results, we conclude that thorough tissue washing is as effective as ELB at removing RBCs from SVF preparations and provides advantages of time savings and diminished risk of potentially toxic substances. Thorough washing also significantly alters the type and relative quantities of WBCs in SVF preparations, significantly reducing neutrophils while increasing the percentage of M2 macrophages.

## Discussion

The major finding of this study is that seemingly trivial steps in tissue processing and cell isolation procedures can significantly alter the final composition of SVF “products.” Specifically, thorough washing of harvested LA tissue prior to enzymatic digestion can significantly alter the amounts and types of BDCs within SVF preparations. Since BDCs can account for as much as 50% of nucleated cells in SVF preparations,^7^ the subtle action of tissue washing may have profound effects on SVF composition. Our findings also show that thorough tissue washing negates the “need” for erythrocyte lysing steps that may release toxic compounds into the extracellular space as well as prolong cell isolation time. We have also demonstrated evidence that the presence and amount of BDCs within the SVF can impact SVF and culture-expanded stromal cell activities such as cell proliferation.

**Figure 5.**
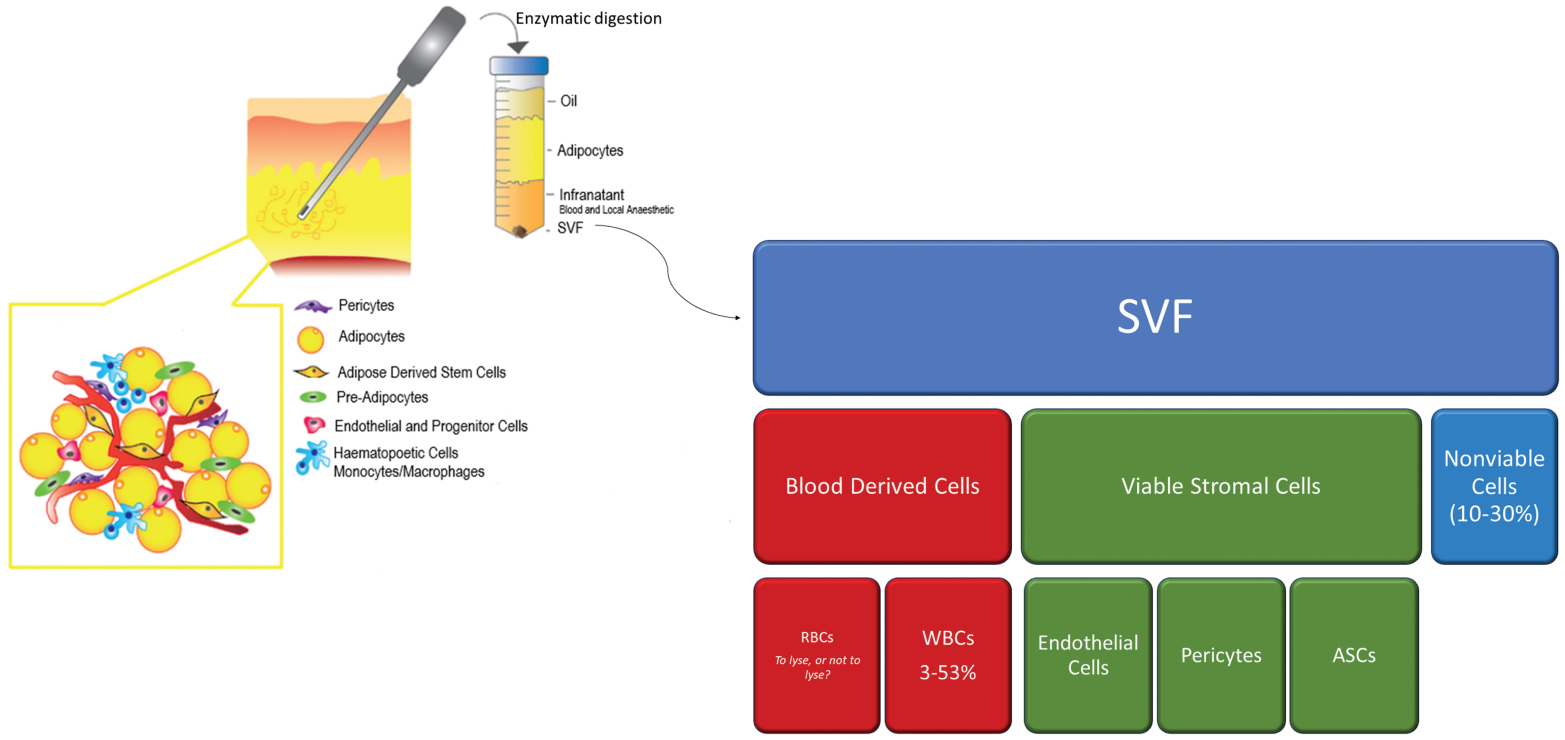
SVF preparations are heterogeneous and their composition and purity may significantly vary depending on a number of factors. In addition to a variety of stromal cell phenotypes, SVF preparations also contain variable amounts of blood-derived cells and non-viable cells. The final composition of SVF is significantly impacted by the extent of tissue washing prior to enzymatic dissociation.

When speaking of the identify and purity of primary uncultured human adipose-derived cell preparations, “SVF” could more accurately be termed the “*H*”-SVF, or hemato-SVF, as it contains both WBCs and RBCs (together BDCs) to various degrees^11^ (Fig. 5). Some WBCs (eg, macrophages) reside normally within the extravascular space as resident cells within adipose tissue but RBCs and the majority of WBCs that are found in SVF preparations result from the trauma, bleeding, and inflammation associated with the harvest procedure.^12,13^ BDCs are often marginalized in the SVF literature, yet account for 30%-80% of the cellular content of SVF preparations reported.^5^

A foundational concept in the regulatory path for all cell therapies is that each cell product must be characterized for cell identity, purity, and potency.^14,15^ Variability in SVF cell composition has long been acknowledged to be largely due to a variety of donor variables and less so to subtle methodological steps common to all adipose-derived cell isolation procedures.^16^ The findings reported herein highlight the fact that SVF therapies produced by different platforms, devices, and methods are NOT necessarily the same; and further, differences and variability between SVF preparations may be related to methodology as well as donor variability.

With this understanding, it is clear that a number of variables can impact the amount of BDCs within SVF preparations including: (1) those inherent to a given donor, (2) the anatomic site and/or duration of the lipo-harvest procedure and the extent of bleeding, (3) whether tumescent solution with epinephrine is used prior to harvest and how long the surgeon waits for vasoconstrictive effect, and (4) the thoroughness of tissue washing prior to collagenase digestion. Since it is practically difficult for a scientist or company to control the first 3 of these variables, it stands to reason that tissue washing prior to enzymatic dissociation is the key and perhaps only step in the isolation process that can impact the number of BDCs that result within the initial SVF pellet. The BDCs that remain after washing and survive enzymatic dissociation are inevitably concentrated into the initial cell pellet. The majority of SVF isolation methods reported in the literature describe the subsequent “removal” of RBCs using one of several available lysis techniques. However, it is unclear when and why this has become the “unwritten standard” in SVF preparation. It may be related to difficulties originally experienced in performing accurate cell counts and viability assessment when using a hemocytometer and trypan blue dye exclusion. However, the advent of fluorescent-based automated cell counters (eg, NucleoCounter, Chemometec) has made this less of a concern.

Our data show that by thorough initial washing of the adipose tissue sample prior to enzymatic dissociation, there is a significant decrease in the number of BDCs in the final product. In unwashed fat, there is a significant burden of RBCs that when treated with lysing buffer is reduced to levels found in well-washed fat. Washing and lysis were found to be equally effective at reducing RBC contamination, but these effects did not compound (washing in conjunction with lysis did not result in improved purity compared to washing or lysis alone). Based on our studies, a thorough washing negates any need or putative benefit obtained from the use of lysing buffer. This may not only reduce the time and steps required for cell product preparation at the point-of-care but also has potential implications for the microenvironment and bioactivity that exists after SVF delivery.

The role of intact RBCs within SVF preparations has not been well studied in the field of adipose-derived cell therapy. Remarkably, culture of both SVF and ASCs in the presence of (intact) RBCs resulted in significantly enhanced SVF cell proliferation compared to cells grown in control media or media with RBC lysate. Moreover, the data suggest a dose-response relationship. Although future work intends to explore the mechanism(s) of this finding, there are several potential explanations. First, RBCs may have simply increased the surface area available for cell growth. It is also possible that the results of the Presto Blue assay were confounded by the RBCs themselves, as there was a direct correlation between RBC concentration in the media and viability, and Presto Blue can be reduced by any metabolically active cell. However, Presto Blue is primarily reduced by mitochondria, which are lacking in RBCs, making this very unlikely.^17^ It is also possible that the RBC stock solutions were contaminated by leukocytes and were the source of additional cell proliferation. However, microscopic visualization of our stock solutions did not reveal any obvious contamination with WBCs; and, even if this were the case, these same cells would have been plated in the lysed RBC cocultures also, since WBCs—as nucleated cells—are not typically destroyed by hypotonic solution. Finally, it is possible that the intact RBCs release one or more mitogenic/trophic factor(s) that act upon one or more of the cell types within the SVF and ASCs.^18-20^

**Figure 6.**
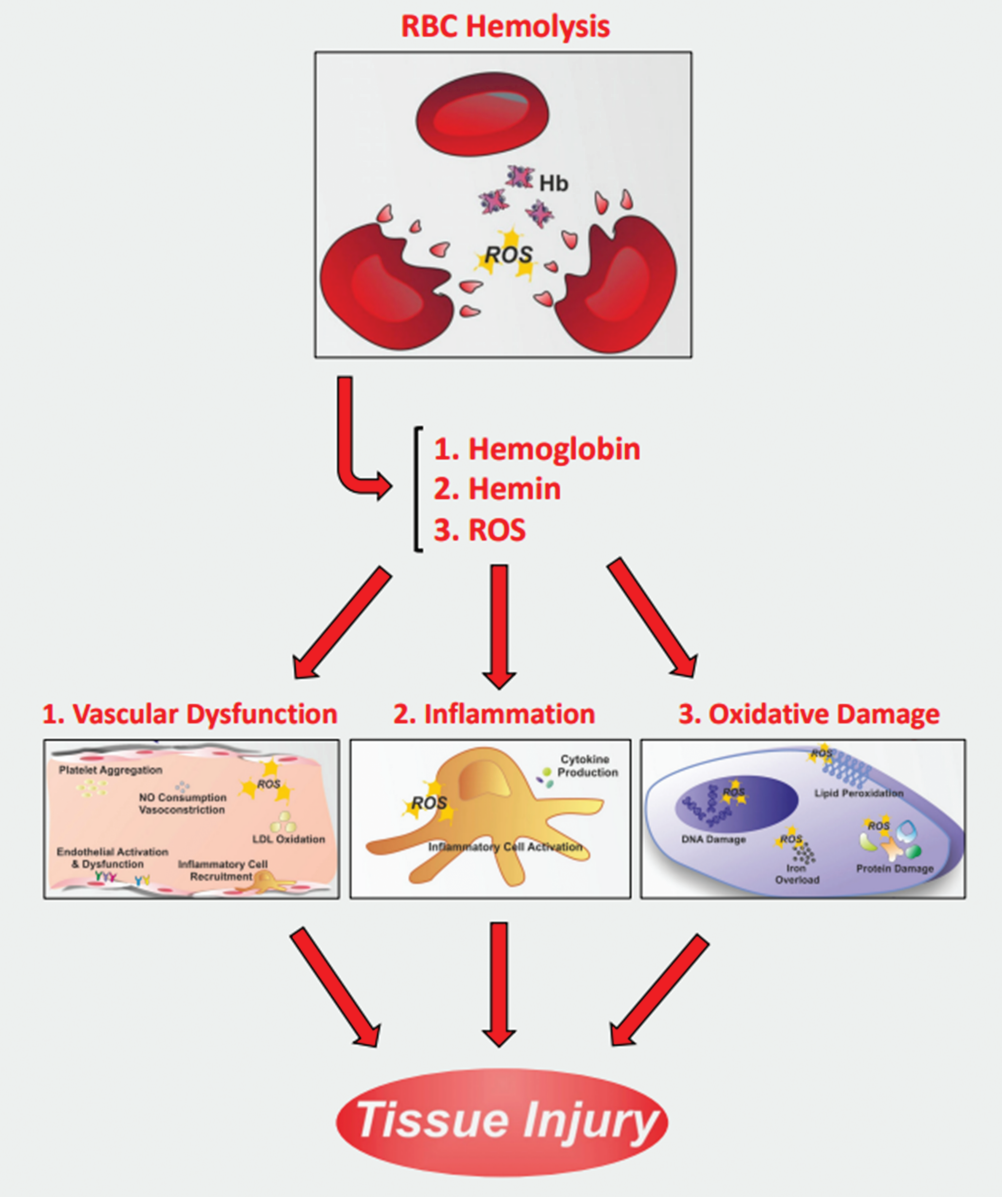
Potential mechanisms of tissue injury after RBC lysis. Inflammation and tissue injury from the lysis of RBCs can result from breakdown products such as hemin and hemoglobin. These compounds are known to cause vascular dysfunction, inflammation, and oxidative damage. While the regenerative properties of SVF may mask some of these effects, optimization of SVF cell therapies may depend on minimizing tissue exposure to these inflammatory agents. (Adapted from Heme in pathophysiology, Frontiers in Pharmacology).^23^

The effect of RBC lysis (RBC lysate) on SVF composition and activity is also poorly explored in the field of adipose-derived cell therapy. When RBCs undergo lysis-free hemoglobin, hemin, and other oxidative stressors are released.^9,21^ It is well documented that these breakdown products of RBCs can produce an inflammatory reaction and require significant biological investment by the immune system to detoxify^9,21,22^ (Fig. 6). In our study, there was a significantly increased concentration of free hemoglobin and hemin detected in SVF and ASC cultures for up to 6 days postlysis that correlates directly with lysed RBC concentration in the conditioned media. However, there was no significant decrease noted in SVF or ASC cell viability/proliferation when cultured in RBC lysate compared to controls. It is possible that the hemoglobin and hemin did not reach a critical threshold necessary to cause a decrease in viability. Or, perhaps, no effects were observed because systemic effector cells that mediate the inflammatory response (eg, neutrophils, macrophages) were lacking in this particular culture model. Conceptually speaking, however, SVF therapies prepared with lysed RBCs have the potential to cause a localized inflammatory response, jeopardizing the viability of surrounding cells that may be beneficial to the patient or altering their response. The question of if and how many RBCs—whether intact or lysed—might negatively or positively affect the biological/therapeutic activity of SVF cell preparations remains an area deserving of further exploration.

Similar to its effect on RBC numbers, our findings demonstrate that thorough washing of initial LA significantly alters the number of WBCs that result in final SVF preparations. Washing reduces the percentage of CD45+ WBCs in final SVF compositions by almost 2-fold, with the greatest reduction (3.5×) specifically found in neutrophil populations (CD45+, CD66b+). Neutrophils are integral to the initial phases of immune-mediated inflammation and, therefore, their presence, in combination with breakdown products from lysed RBCs, may be associated with unintended and/or undesired therapeutic effects at their site of delivery. Further research is needed to explore this possibility. Lysis had no effect on these cell types and neither washing nor lysis had any meaningful effect on the percentage of other viable nucleated cells within SVF preparations or on circulating macrophages (CD45+, CD64+), M1 polarized macrophages (CD86+), or monocytes (CD14+).

Interestingly, and somewhat unexpected, was the finding that washing significantly increased the percentage of CD 206+ macrophages in SVF products. CD 206 is often cited as a marker of M2 macrophages, which are associated with tissue remodeling and repair. Explanations for this finding include the potential enrichment of tissue resident M2 cells relative to a smaller WBC population that has resulted from more thorough washing. Alternatively, this finding may reflect differences in CD 206+ integrins which make them more resistant to dislodging by washing. This, in turn, may reflect differences between tissue-resident and acute infiltrating blood-derived leukocytes, but more study is needed. In any case, it is possible that the significant depletion of neutrophils and RBCs through washing, along with the relative enrichment of M2 macrophages and the avoidance of RBC lysis (and release of associated inflammatory mediators) is associated with clinically meaningful differences in the safety and/or efficacy of different SVF “products.” Based on the findings of this work, we propose that translational efforts in the field would benefit by a better understanding of the impact of RBCs, WBCs and nonviable cells on the in vivo therapeutic activity of SVF therapies.

## Conclusion

We provide evidence that intact RBCs improve SVF and ASC growth in vitro at certain concentrations, and future work may explore the mechanism of this finding. Tissue washing and RBC lysis are equally effective at reducing RBC “contamination,” but these effects are not synergistic (washing in conjunction with lysis did not result in improved purity compared to washing or lysis alone). Conversely, washing exhibits superior CD45+ WBC purification compared to lysis, but again the effect does not compound. This same pattern holds true for CD66b+ (neutrophil) and CD206+ (M2 Macrophage) cells. Washing and lysis have no effect on CD64+ (macrophage), CD86+ (M1 Macrophage), or CD14+ (monocyte) lineage concentrations. Broadly, these data exemplify how different, seemingly mundane tissue processing steps can significantly influence BDC subpopulations present within SVF. Future research should examine how BDCs and/or nonviable cells within SVF preparations confer detectable and modifiable effects on in vitro and in vivo SVF bioactivity. Exploring the effects of washing and lysis on BDC populations/products is part of the broader necessity to standardize SVF purification procedures for widespread therapeutic use that is both reproducible and predictable.

## Supplementary Material

szad025_suppl_Supplementary_Materials

## Data Availability

The data that support the findings of this study are available from the corresponding author upon reasonable request.
