# Supplementary material for: Extent of Tissue Washing Can Significantly Alter the Composition of Adipose-Derived Stromal Vascular Fraction Cell Preparations: Implications for Clinical Translation
Source: Stem Cells Transl Med. 2023 Jun 3;12(6):391–9. doi: 10.1093/stcltm/szad025 (PMC10267576; doi:10.1093/stcltm/szad025)
Supplement: szad025_suppl_Supplementary_Materials [file szad025_suppl_supplementary_materials.docx]

Supplemental Figures


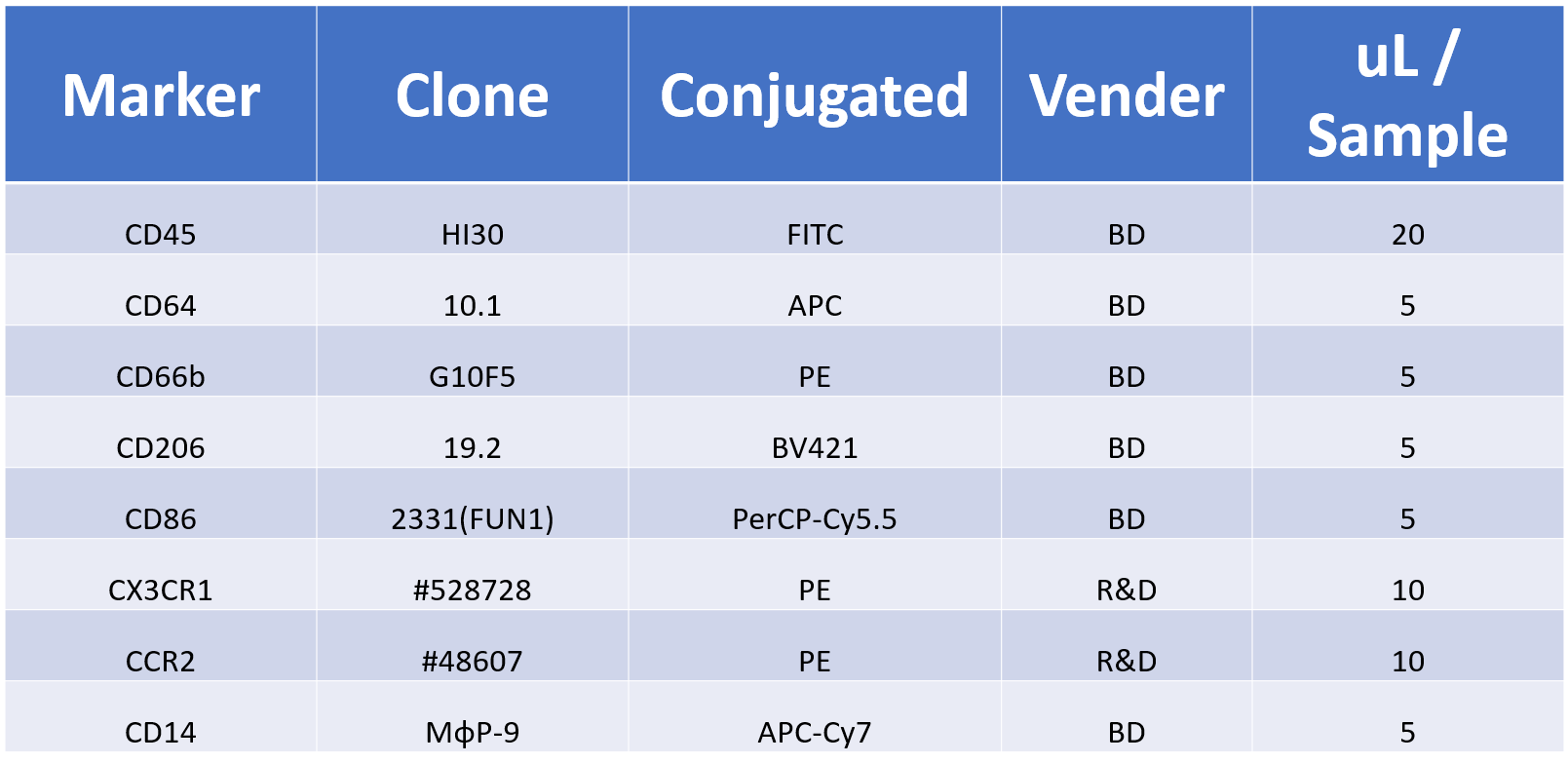


**Supplemental Table 1. Antibodies for Flow Cytometry.** The above antibodies were utilized for staining samples and run through a BD FacsCanto II flow cytometry unit immediately after the staining procedure was completed.


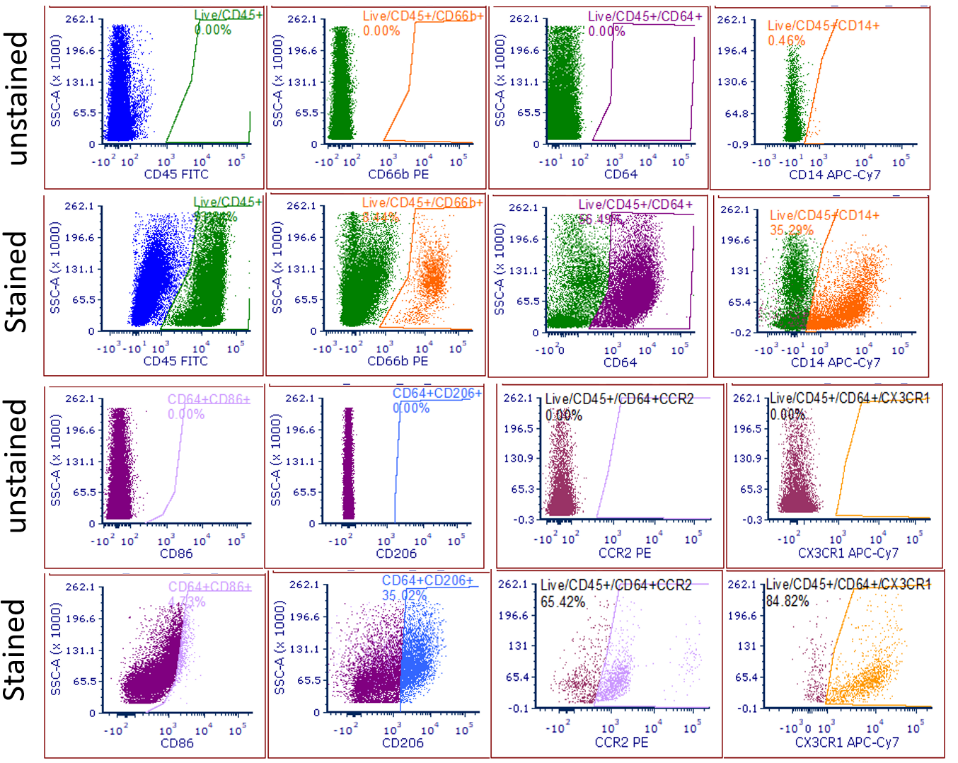

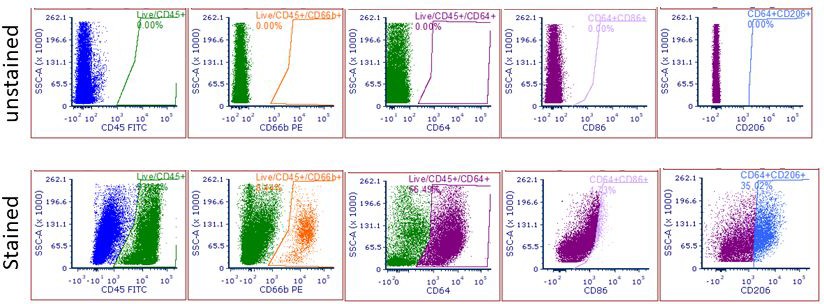


**Supplemental Figure 1. Flow Cytometry Results Interpretation.** Results were gated and analyzed based on the proportions of target cells to one another. All samples were analyzed in the same manner.


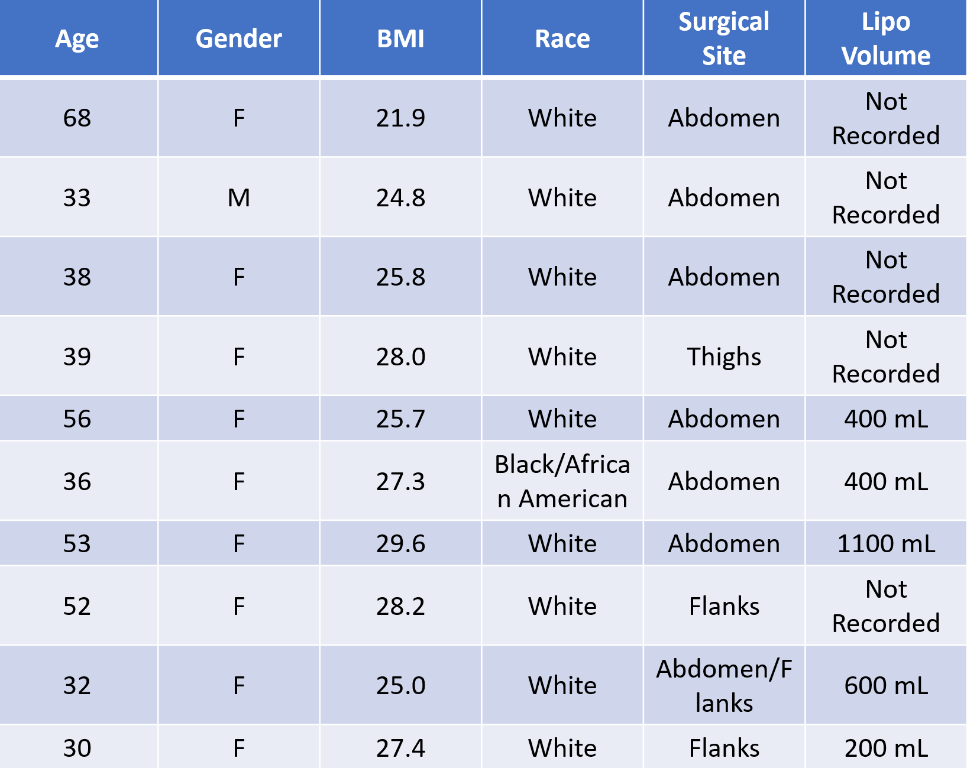


**Supplemental Table 2. Summary Demographics of Tissue Donors.**


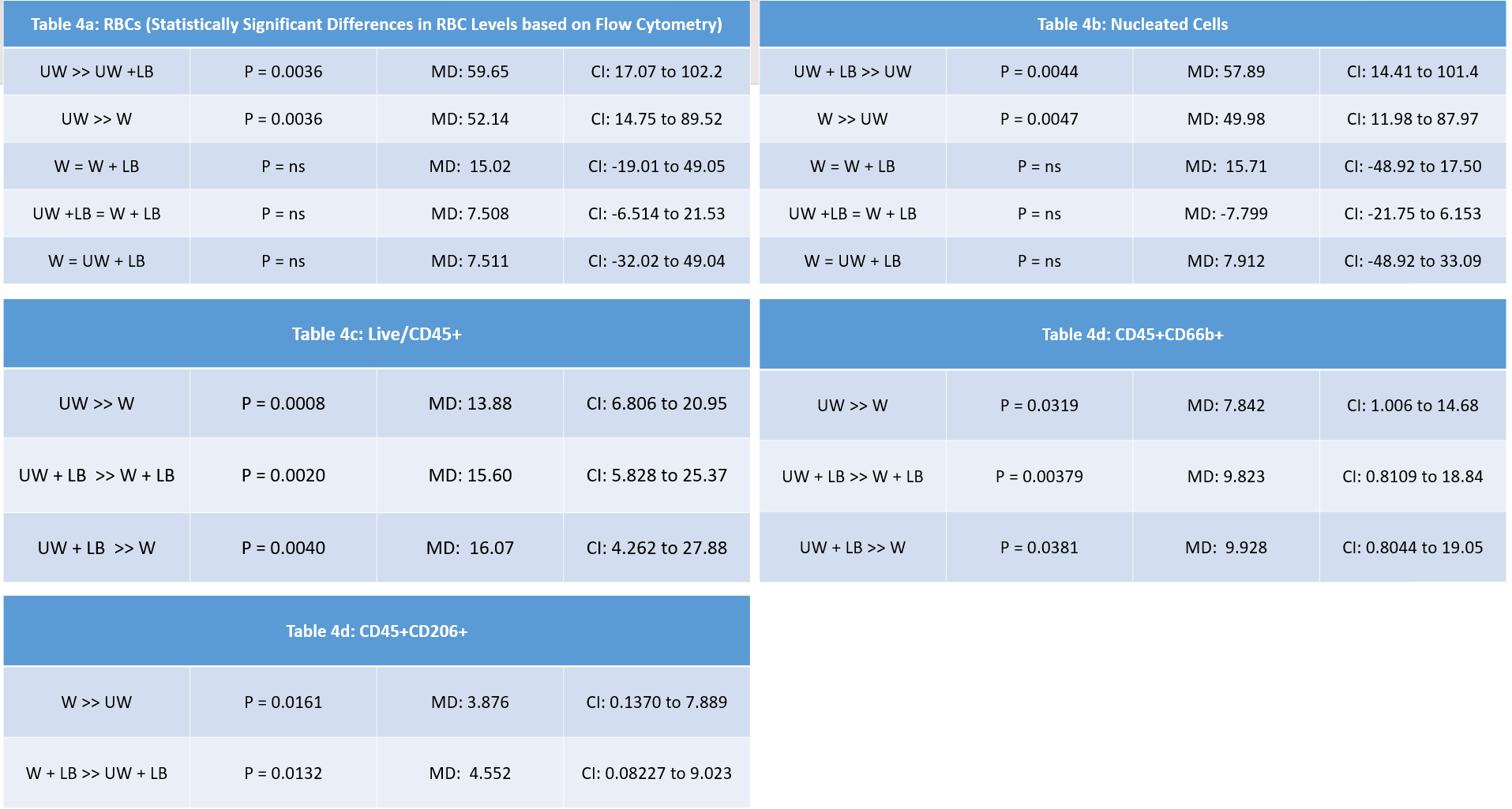


**Supplemental Table 3. Statistical Significance Between Extraction Methods.** We utilized ANOVA tests with subsequent Dunnett’s to determine significance between Washed (W), Unwashed (UW), and Lysis Buffer (LB). Above is a tabular representation of the differences that we found utilizing this method including Mean Deviations (MD) and Confidence Intervals (CI). These differences illustrate how different processing methods target different cell populations.
